# Supplementary material for: Inhibition of microRNA-33b in humanized mice ameliorates nonalcoholic steatohepatitis
Source: Life Sci Alliance. 2023 Jun 1;6(8):e202301902. doi: 10.26508/lsa.202301902 (PMC10235800; doi:10.26508/lsa.202301902)
Supplement: Supplementary file 1 [file LSA-2023-01902_TableS1.docx]

| **Supplementary table 1.** Serum data of miR-33^fl/fl^ KI and *Ayu1*-Cre/miR-33b^fl/fl^ KI mice | | | | |
| --- | --- | --- | --- | --- |
|  |  |  |  |  |
|  | **miR-33b^fl/fl^ KI** | ***Ayu1*-Cre/miR-33b^fl/fl^ KI** |  |  |
| TP (g/dL) | 4.44 ± 0.07 | 4.56 ± 0.07 |  |  |
| ALB (g/dL) | 3.04 ± 0.07 | 3.20 ± 0.09 |  |  |
| AST (IU/L) | 66.8 ± 10.7 | 60.0 ± 4.5 |  |  |
| ALT (IU/L) | 28.0 ± 2.6 | 35.6 ± 5.4 |  |  |
| LDH (IU/L) | 798.8 ± 65.7 | 741.6 ± 88.1 |  |  |
| T-BIL (mg/dL) | 0.088 ± 0.019 | 0.068 ± 0.030 |  |  |
| T-Cho (mg/dL) | 68.0 ± 3.3 | 104.8 ± 8.7 | ^**^ |  |
| LDL-C (mg/dL) | 9.6 ± 1.8 | 9.2 ± 0.5 |  |  |
| HDL-C (mg/dL) | 42.4 ± 2.0 | 63.6 ± 6.8 | ^*^ |  |
| TG (mg/dL) | 76.0 ± 9.5 | 70.0 ± 5.4 |  |  |
| NEFA (μEq/L) | 947.6 ± 122.7 | 892.4 ± 70.5 |  |  |
| Male mice were fed NC. Sample were obtained at the age of 8 weeks. | | | | |
| Values are the mean ± S.E.M., n = 5 each, ^*^p<0.05, ^**^p<0.01, unpaired t-test. | | | |  |
