# Supplementary material for: Inhibition of microRNA-33b in humanized mice ameliorates nonalcoholic steatohepatitis
Source: Life Sci Alliance. 2023 Jun 1;6(8):e202301902. doi: 10.26508/lsa.202301902 (PMC10235800; doi:10.26508/lsa.202301902)
Supplement: Supplementary file 2 [file LSA-2023-01902_TableS2.docx]

| **Supplementary table 2.** Serum data of miR-33^fl/fl^ KI and *Alb*-Cre/miR-33b^fl/fl^ KI mice | | | | |
| --- | --- | --- | --- | --- |
|  |  |  |  |  |
|  | **miR-33b^fl/fl^ KI** | ***Alb*-Cre/miR-33b^fl/fl^ KI** |  |  |
| TP (g/dL) | 4.32 ± 0.07 | 4.18 ± 0.07 |  |  |
| AST (IU/L) | 53.4 ± 2.4 | 47.5 ± 4.7 |  |  |
| ALT (IU/L) | 25.8 ± 2.0 | 19.7 ± 2.1 |  |  |
| ALP (IU/L) | 216.9 ± 31.0 | 167.3 ± 9.6 |  |  |
| T-BIL (mg/dL) | 0.102 ± 0.018 | 0.100 ± 0.013 |  |  |
| T-Cho (mg/dL) | 61.2 ± 0.73 | 91.2 ± 6.3 | ^**^ |  |
| LDL-C (mg/dL) | 4.2 ± 0.7 | 6.7 ± 0.4 | ^*^ |  |
| HDL-C (mg/dL) | 37.2 ± 1.5 | 53.8 ± 4.4 | ^**^ |  |
| TG (mg/dL) | 60.6 ± 5.0 | 59.3 ± 4.7 |  |  |
| NEFA (μEq/L) | 792.6 ± 51.6 | 756.7 ± 87.2 |  |  |
| Male mice were fed NC. Sample were obtained at the age of 8 weeks. | | | | |
| Values are the mean ± S.E.M., n = 5–6 each, ^*^p<0.05, ^**^p<0.01, unpaired t-test. | | | | |
