# Supplementary material for: Inhibition of microRNA-33b in humanized mice ameliorates nonalcoholic steatohepatitis
Source: Life Sci Alliance. 2023 Jun 1;6(8):e202301902. doi: 10.26508/lsa.202301902 (PMC10235800; doi:10.26508/lsa.202301902)
Supplement: Supplementary file 3 [file LSA-2023-01902_TableS3.docx]

| **Supplementary table 3.** Serum data of miR-33^fl/fl^ KI and *LysM*-Cre/miR-33b^fl/fl^ KI mice | | | | |
| --- | --- | --- | --- | --- |
|  |  |  |  |  |
|  | **miR-33b^fl/fl^ KI** | ***LysM*-Cre/miR-33b^fl/fl^ KI** |  |  |
| TP (g/dL) | 4.20 ± 0.08 | 4.35 ± 0.05 |  |  |
| ALB (g/dL) | 2.90 ± 0.06 | 3.00 ± 0.00 |  |  |
| AST (IU/L) | 54.0 ± 6.1 | 41.0 ± 2.6 |  |  |
| ALT (IU/L) | 26.0 ± 3.6 | 16.0 ± 1.4 | ^*^ |  |
| LDH (IU/L) | 301.5 ± 39.1 | 293.0 ± 30.0 |  |  |
| T-BIL (mg/dL) | 0.100 ± 0.034 | 0.075 ± 0.010 |  |  |
| T-Cho (mg/dL) | 68.0 ± 4.9 | 68.5 ± 4.1 |  |  |
| LDL-C (mg/dL) | 7.0 ± 1.9 | 7.5 ± 1.0 |  |  |
| HDL-C (mg/dL) | 40.0 ± 3.6 | 41.0 ± 1.3 |  |  |
| TG (mg/dL) | 26.4 ± 0.4 | 24.5 ± 0.5 |  |  |
| NEFA (μEq/L) | 703.0 ± 64.6 | 609.0 ± 71.0 |  |  |
| Male mice were fed NC. Sample were obtained at the age of 8 weeks. | | | | |
| Values are the mean ± S.E.M., n = 4 each, ^*^p<0.05, unpaired t-test. | | |  |  |
