# Supplementary material for: Inhibition of microRNA-33b in humanized mice ameliorates nonalcoholic steatohepatitis
Source: Life Sci Alliance. 2023 Jun 1;6(8):e202301902. doi: 10.26508/lsa.202301902 (PMC10235800; doi:10.26508/lsa.202301902)
Supplement: Supplementary file 4 [file LSA-2023-01902_TableS4.docx]

| **Supplementary table 4.** Serum data of miR-33^fl/fl^ KI and *LysM*-Cre/miR-33b^fl/fl^ KI mice fed a 45% HFD | | | | |
| --- | --- | --- | --- | --- |
|  |  |  |  |  |
|  | **miR-33b^fl/fl^ KI** | ***LysM*-Cre/miR-33b^fl/fl^ KI** |  |  |
| TP (g/dL) | 4.48 ± 0.11 | 4.63 ± 0.17 |  |  |
| ALB (g/dL) | 2.93 ± 0.11 | 3.08 ± 0.06 |  |  |
| AST (IU/L) | 96.8 ± 25.3 | 97.5 ± 14.3 |  |  |
| ALT (IU/L) | 82.8 ± 32.5 | 86.5 ± 17.6 |  |  |
| ALP (IU/L) | 171.0 ± 13.3 | 216.3 ± 17.8 |  |  |
| LDH (IU/L) | 799.8 ± 153.1 | 662.0 ± 110.7 |  |  |
| T-BIL (mg/dL) | 0.088 ± 0.017 | 0.078 ± 0.006 |  |  |
| TBA (μmol/L) | 2.25 ± 0.63 | 2.25 ± 0.95 |  |  |
| T-Cho (mg/dL) | 97.5 ± 8.3 | 112.3 ± 13.5 |  |  |
| LDL-C (mg/dL) | 6.8 ± 0.5 | 8.0 ± 1.8 |  |  |
| HDL-C (mg/dL) | 56.3 ± 1.5 | 58.3 ± 3.1 |  |  |
| TG (mg/dL) | 31.3 ± 2.6 | 38.0 ± 3.5 |  |  |
| NEFA (μEq/L) | 506.0 ± 56.1 | 498.8 ± 31.3 |  |  |
| Male mice were fed a 45% HFD from the age of 8 weeks for 12 weeks. | | |  |  |
| Values are the mean ± S.E.M., n = 4 each, unpaired t-test. | | |  |  |
