# Supplementary material for: Inhibition of microRNA-33b in humanized mice ameliorates nonalcoholic steatohepatitis
Source: Life Sci Alliance. 2023 Jun 1;6(8):e202301902. doi: 10.26508/lsa.202301902 (PMC10235800; doi:10.26508/lsa.202301902)
Supplement: Supplementary file 5 [file LSA-2023-01902_TableS5.docx]

| **Supplementary table 5.** Primers for genotyping and product size | | |
| --- | --- | --- |
|  |  |  |
| Primer | Sequence | Product (bp) |
| miR-33b WT and KI (S) | ATGGATTTACCTCAGTTTTAACGAC |  |
| miR-33b WT (A) | CATCACTGAAGCACTGCATCTGC |  |
| miR-33b KI (A) | AAGTGGATCCAGAATTCGTGA | WT/KI 199/491 |
| miR-33b^flox^ (S) | TCTTCTCCCGAGCTCTCTTACTCTCA |  |
| miR-33b^flox^ (A) | TTATTCCCCACGATGGTTAGGTAGGC | WT/floxed 256/542 |
| Cre (S) | GAACCTGATGGACATGTTCAGG |  |
| Cre (A) | AGTGCGTTCGAACGCTAGAGCCTGT | 320 |
| Myogenin (internal control) (S) | TTACGTCCATCGTGGACAGC |  |
| Myogenin (internal control) (A) | TGGGCTGGGTGTTAGCCTTA | 250 |
| Southern blotting primer (S) | AGTAAAATTCTCCTCAATGAACGTG |  |
| Southern blotting primer (A) | CAGTAGGTGACATTGTGATTGATCT | 468 |
