# Supplementary material for: Inhibition of microRNA-33b in humanized mice ameliorates nonalcoholic steatohepatitis
Source: Life Sci Alliance. 2023 Jun 1;6(8):e202301902. doi: 10.26508/lsa.202301902 (PMC10235800; doi:10.26508/lsa.202301902)
Supplement: Supplementary file 6 [file LSA-2023-01902_TableS6.docx]

| **Supplementary table 6.** Primers used in quantitative real-time PCR in this study | | | |
| --- | --- | --- | --- |
|  |  |  |  |
| Species | Gene | Sequence (S) | Sequence (A) |
| Mouse | *Srebf1* | TAGAGCATATCCCCCAGGTG | GGTACGGGCCACAAGAAGTA |
|  | *Srebf2* | GTGGAGCAGTCTCAACGTCA | TGGTAGGTCTCACCCAGGAG |
|  | *Abca1* | AACAGTTTGTGGCCCTTTTG | AGTTCCAGGCTGGGGTACTT |
|  | *Crot* | GACGGTCAAATCCTTTTCCA | TACTTTTACCACGGCCGAAC |
|  | *Cpt1a* | GATCTACAATTCCCCTCTGCTCT | TAGAGCCAGACCTTGAAGTAACG |
|  | *Prkaa1* | TGATCAGCACTCCGACAGAC | TCTCTGGCTTCAGGTCCCTA |
|  | *Sirt6* | GGCTACGTGGATGAGGTGAT | GGCTCAGCCTTGAGTGCTAC |
|  | *Atp8b1* | TACGGTCATGGTGGACAGAA | AATGGCAAGGACGTTGTAGG |
|  | *Abcb11* | GGCTTGCTACAGATGCTTCC | GCCAAAAAGGGGAAGAAGAC |
|  | *Tnf* | CCAGACCCTCACACTCAGATC | CACTTGGTGGTTTGCTACGAC |
|  | *Col1a1* | GCCAAGAAGACATCCCTGAAG | TCATTGCATTGCACGTCATC |
|  | *Acta2(αSma)* | TCCCTGGAGAAGAGCTACGAACT | GATGCCCGCTGACTCCAT |
|  | *Il6* | ACCACGGCCTTCCCTACTTC | AGATTGTTTTCTGCAAGTGCATCA |
|  | *Cd68* | TTCTGCTGTGGAAATGCAAG | AGAGGGGCTGGTAGGTTGAT |
|  | *F4/80* | TCCAGAAGGCTCCCAAGGATA | GGGCACTTTTGTTCTCACAGGTA |
|  | *18S* | CGCGGTTCTATTTTGTTGGT | AGTCGGCATCGTTTATGGTC |
| Human | *ABCA1* | GTCCTCTTTCCCGCATTATCTGG | AGTTCCTGGAAGGTCTTGTTCAC |
|  | *CPT1A* | CATCATCACTGGCGTGTACC | TTGGCGTACATCGTTGTCAT |
|  | *CROT* | GCTTCACCCGGATACGTTTA | CGCTCACGAAGATTGACAGA |
|  | *COL1A1* | TTGACCAACCGAACATGACC | TTCAAGCAAGTGGACCAAGC |
|  | *ACTA2(αSMA)* | GAAGGAATAGCCACGCTCAG | TTCAATGTCCCAGCCATGTA |
|  | *POSTN* | AAACCATCGGAGGCAAACAG | TCGCGGAATATGTGAATCGC |
|  | *ACTB* | AGGCACTCTTCCAGCCTTCC | GCACTGTGTTGGCGTACAGG |
